# Dermatologic aspects of ectodermal dysplasias

**DOI:** 10.1186/1746-160X-8-S1-I4

**Published:** 2012-05-25

**Authors:** Peter H Itin

**Affiliations:** 1 Department of Dermatology, University of Basel, Switzerland

## 

Ectodermal dysplasias are a large group of heterogeneous heritable conditions characterized by congenital defects of ectodermal structures and their appendages: hair (hypotrichosis, partial or total alopecia), nails (dystrophic, hypertrophic, abnormally keratinized), teeth (enamel defect or absent) and sweat glands (hypoplastic or aplastic). The ectodermal dysplasias, as a rule, are not pure “one-layer diseases”. Mesodermal and occasionally endodermal dysplasias may coexist. Embryogenesis occurs in distinct tissue fields and specific interactions among the germ layers that may lead to a wide range of ectodermal dysplasias exist when genes important for development are mutated or otherwise altered in expression. Of the approximately 200 different ectodermal dysplasias, about 50 have been characterized at the molecular level with identification of the causative gene. Modern molecular genetics will increasingly elucidate the basic defects of the different syndromes and yield more insight into the regulatory mechanisms of embryogenesis.

This lecture focuses on the fact that with molecular diagnosis it is possible to diagnose oligosymptomatic forms of ectodermal dysplasia. These are much more common than earlier anticipated. Cutaneous key features which give hints for the presence of some type of ectodermal dysplasia will be presented.

